# Reply: Blunted Exercise Response in Cancer Survivors: A Consequence of the Heart or Peripheral Muscle?

**DOI:** 10.1016/j.jaccao.2025.09.001

**Published:** 2025-09-25

**Authors:** Tormod S. Nilsen, Sara Hassing Johansen

**Affiliations:** Norwegian School of Sport Sciences, Oslo, Norway

We thank Drs Xu and Zhao for their encouraging comments on the CAUSE (Cardiovascular Survivors Exercise) trial. We support their hypothesis that skeletal muscle toxicities from treatment contribute to the blunted exercise response we observed in breast cancer survivors (BCSs) compared with cancer-naive women. As stated by Drs Xu and Zhao, anthracycline toxicity may affect all stages of the O_2_ cascade, and the limiting factors for physiological adaptations to exercise therapy remain debated. Interestingly, Xu and Zhao point to skeletal muscle mitochondria dysfunction as a plausible limiting factor for improved cardiorespiratory fitness. Supporting this, we have published findings from genome-wide methylation analysis in skeletal muscle biopsies collected from a subgroup of participants from the CAUSE trial.^1^ Our findings showed increased DNA hypermethylation in BCSs (∼10 years post-treatment) compared with the cancer-naive women at baseline, despite similar physical activity levels. A hypermethylated DNA signature is associated with increased age,^2^ which indicates that epirubicin-treated BCSs possess muscles with a more aged profile compared with cancer-naive peers. Importantly, aerobic exercise promoted changes that reversed the methylome signature toward the signature observed in the cancer-naive women.^1^ Whether the hypermethylated DNA signature impairs mitochondrial adaptations is under investigation in our laboratory,^1^ and further insight into limiting factors for cardiorespiratory fitness responses from the CAUSE trial will follow.

We support Drs Xu and Zhao’s call for increased focus on “myo-oncology.” Muscle loss and dysfunction are well-known side effects of several anticancer therapies, but the discussion is often limited to cancer cachexia. We do not intend to undermine the importance of cancer cachexia, which is associated with poor survival in some patient populations, but rather encourage research into the muscle loss and dysfunction occurring on a larger scale among cancer patients, impairing survivorship. In addition, the majority of knowledge on skeletal muscle side effects of anticancer therapies is derived from preclinical studies. Although important for the mechanistic understanding of side effects from different treatment modalities, findings in preclinical models are difficult to replicate in human studies. Additionally, muscle loss and dysfunction may not be explained by muscle cell signaling pathways alone in clinical settings but are also affected by other factors such as physical inactivity, suboptimal nutrition, and nausea.^3^ Hence, the landscape of muscle toxicity in cancer is inherently complex, and multidisciplinary approaches are needed to identify challenges and opportunities for improved survivorship. We therefore welcome “myo-oncology” as a novel subdiscipline within oncology to increase the focus on skeletal muscle health, ultimately leading to improved survivorship.
